# A new perspective to an old problem – Mobilizing research into policy and practice using an arts and health case study

**DOI:** 10.3389/fpubh.2024.1392146

**Published:** 2024-04-17

**Authors:** Christina Davies, Melanie Pescud, Susan Maury, Denise Sullivan

**Affiliations:** ^1^Centre for Arts, Mental Health and Wellbeing WA, School of Allied Health and School of Humanties, The University of Western Australia, Crawley, WA, Australia; ^2^Policy, Strategy, and Impact Group, VicHealth, Victorian Health Promotion Foundation, West Melbourne, VIC, Australia; ^3^Chronic Disease Prevention Directorate, Public and Aboriginal Health Division, WA Department of Health, East Perth, WA, Australia

**Keywords:** arts, health policy, mental health, health promotion, knowledge mobilization, translation

## Introduction

**Figure 1:**
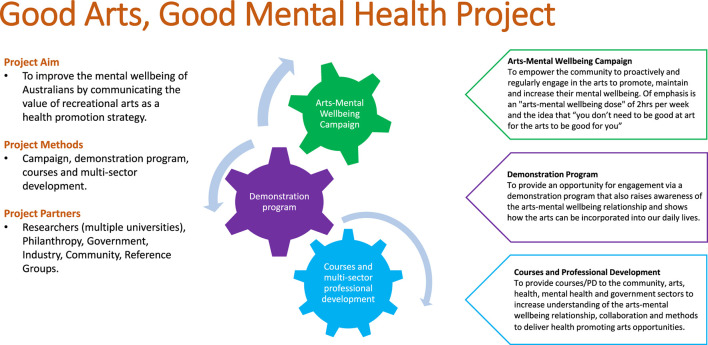
Good arts, good mental health project summary.

This opinion piece provides insights essential to developing trust and collaboration with stakeholders that make for innovative research mobilization into policy and practice. We summarize key strategies from the field of knowledge mobilization, illustrated by the Good Arts, Good Mental Health^®^ project (GAGMH) as a case study (Figure 1). The GAGMH is based at the University of Western Australia and has developed/leveraged partnerships with six universities, the community, arts-mental health reference groups and 30 government, industry, and philanthropic partners. GAGMH is evidence-based (1–8), award winning, and aims to improve the mental wellbeing of Australians by communicating the value of recreational arts via a public health mass media campaign, demonstration programs, and the delivery of multi-sector courses and professional development to the community, arts, health, and local government sectors. With a community endorsed catch-cry of “*You don't have to be good at art for the arts to be good for you*,” GAGMH is an example of knowledge mobilization in the emerging health promotion area of Arts and Health (2). Arts and Health is defined as “*the use of the arts to promote, maintain, or improve health and wellbeing; and/or the introduction of the arts into a setting to enhance the health environment (e.g., paintings in hospital rooms, music in waiting rooms*)” (2). Recreational arts engagement is the arts people take-part in for enjoyment, entertainment, socially, or as a hobby (e.g., listening to music, reading books, singing, coloring, photography, concerts) and can occur in a variety of settings including the home, schools, work, community centers, concert halls, etc. (2).

Globally, mental health issues are increasing (9). Given the persuasive evidence that recreational arts engagement enhances mental wellbeing (1, 7, 8), including a quantification that links 2 h of arts per week to mental wellbeing (i.e., the “Arts dose”) (3), it is time to innovate using knowledge mobilization to more effectively utilize this low cost, person-centered, non-pharmacological method for prevention. Knowledge mobilization is a strategy for improving public health policy and practice. It refers to the multi-directional transfer of information, informed by diverse sources, tailored to specific audiences and a variety of ways of thinking (10, 11). Our understanding of the way knowledge moves has changed from simple, linear models to systems thinking approaches that consider multi-directionality, complexity, and reciprocal partnerships (10). Recognizing the intricacies of the public health policy/practice landscape (12), and incorporating both theory and our experience of mobilizing research that informs GAGMH, we present two themes for readers to consider (a) awareness; (b) attitudes and action.

## Knowledge mobilization and GAGMH

### Awareness

A first step to affecting change is a deep understanding of the social and political settings in which policy/practice operate. This includes an appreciation of the dynamism of policy agendas—especially its informants, structures, and facilitators. A first step to influencing policy/practice is an awareness of the larger system you are attempting to change (12, 13). Using GAGMH as an example, this includes:

a) **The sector(s) who could/should/may utilize your research**. For GAGMH, this includes health, mental health, arts, and government sectors (to name a few).
b) **The target groups that may benefit from your research**. The target group for GAGMH is the general population and sub-groups within the population e.g., young people, older adults, low-income. Using processes ranging from consultation to co-design (14), it is important to engage with and involve members of your target group to understand their experiences, priorities, needs, barriers, and how they may benefit from changes to policy and/or practice. GAGMH has a community reference group and when mobilizing research into a campaign, courses/professional development and a demonstration program, the general population have had a platform to share their experiences via submissions, surveys, focus groups, and interviews.
c) **Informants that guide your research**. GAGMH is informed by a multi-university team, the community, reference groups, and 30 government, industry, and philanthropic partners who we meet with quarterly. Surveys, focus groups, and interviews have also been used to generate informant insights.
d) **Existing policies, new policies, and policy cycles**. Windows of opportunity to influence policy open when three streams connect, i.e., policy, problems, and politics (15). In terms of “policy,” the research underpinning GAGMH is cited in “Connected Lives—Creative Solutions to the Mental Health Crisis” policy document (16), and “Revive,” Australia's cultural policy (“Policy”) (17). The window of opportunity emerged via a consultation and submission process in the months prior the development of both documents. For Government to be made aware of the solutions your research provides, it is worth writing formal submissions and participating in public consultations (e.g., inquiries, commissions, plans, policy development) (18). In addition, to influence policy, you need to be aware of policy cycles. Most government policies run on 3-, 4- or 5-year cycles. It is possible to influence policy when they are being developed or reviewed which usually occurs 1 year before the end of the policy cycle. Elections, senate inquiries, and royal commissions provide an opportunity for policy development and change. Regarding the “problem,” the “Connected Lives” consultation report outlines ways to address the current mental health crisis by utilizing the arts, culture, and creativity; while “Revive” outlines a plan to revitalize the arts, strengthen culture, wellbeing, and social connectedness. It is important to identify the relationships between election commitments, current issues, and your research, e.g., GAGMH relates to current “hot-button” issues of community mental health and arts revitalization for engagement and wellbeing. Considering “politics,” this builds on the legacy of the Whitlam government that acknowledged the “vital role” of the arts in wellbeing, national identity, social connection, and economic success (17).
e) **Appropriate communication**. This involves knowing your audience, speaking their language, respecting their values, adhering to their communication styles, and learning how to frame/reframe issues (13, 19). If your audience prefers qualitative information, then case studies, personal connection, and storytelling may be preferred. In comparison, an audience that prefers quantitative information may expect research that includes statistical significance, effect size, generalizability, and applicability (20). If you recognize you don't have the communication style or language that your audience prefers, it is beneficial to find a mentor, translator, or “boundary spanner” to guide you (21). This is especially the case when working cross-sectorally or with an audience you are unfamiliar with. A mentor, translator, or boundary spanner will help you understand the values, pace of work, timelines, ways to frame issues, and decision-making drivers of that sector/audience, e.g., GAGMH mentors include individuals from industry, philanthropy, and government departments including health, mental health, and the arts.
f) **Stakeholders with the remit to effect change**. You need to identify which of your stakeholders have the power to address the change you are advocating for, and who may lobby against your work. It is also important to know who in government or industry may be focused on a similar issue and who has the remit to approve, prioritize, and implement your work (12). Your stakeholders should include policy end users, advocates, and people who write, or update policy/practice. You may wish to meet with, present to, or email your research to relevant people including Ministers, directors, policy officers, and advocates. Understanding your existing networks and the networks of your stakeholders is also useful when mobilizing knowledge (22). For example, GAGMH outcomes as well as arts-health policy/practice in Australia have been enhanced through the power and influence of stakeholders that cluster within the network of health professionals, artists, researchers, industry, philanthropists, media, and government.

### Attitudes and actions

When thinking about policy/practice, your attitudes and actions toward knowledge mobilization are important.

a) **Cultivate actions of a change maker and be the “go-to” person**. To influence policy and practice you need to be clear on the change you seek to make (12, 13, 23). We suggest choosing one aim as your focus (which may have several objectives) and cultivating a strong resolve to work toward that aim. Regarding “action,” your research, teaching, and service roles should align with this aim. As above, the aim of GAGMH is to improve the mental wellbeing of Australians by communicating the value of recreational arts as a health promotion strategy. Therefore, the “research” to guide this project focused on defining, qualifying, and quantifying the arts-mental health relationship (1–5, 24), “teaching” stakeholders about research findings via face-to-face and online courses, professional development, conferences, and community presentations; and “service roles” including engaging with the media, a strong social media presence, and contributing to boards and advisory groups. You need to strive to become the “go-to-person” in your area of expertise that the media, industry, and government approach. To get your foot in the door, a mentor with established networks may be advantageous for initiating introductions and attending meetings (13). It is also suggested that you share your research with government and industry and that you meet informally (e.g., for coffee) with policy officers, policy makers, and advocates to understand their viewpoints and the issues they are most interested in resolving. If your research is relevant and aligns with an issue that government/industry are trying to understand, improve, or solve, it is more likely to be used.
b) **Practice a steadfast attitude and develop respectful collaborations with key stakeholders**. Be steadfast in your attitude to see your research mobilized. While the “wave” for your research may not happen immediately, it will happen in time if you harness the power of advocacy, the media, social media, and develop respectful collaborations with key stakeholders. The “wave” for GAGMH took over 10 years from initial idea, to conducting and mobilizing the research, to enhancing knowledge mobilization efforts by collaborating with philanthropy, government, advocates, the community, and industry. While a steadfast attitude is needed to achieve your aim, at times, you may need to cast your ego aside and see advice, criticism, or rejection of your work as an opportunity to learn, adapt, strengthen your argument, reframe your approach, and evolve your thinking. Given that university-based research is often “theory driven,” respecting the practical, “issue relevant” knowledge and feedback from stakeholders is paramount to a respectful collaboration. For example, when writing a paper or grant, policy end users, advocates, government, and industry should be active collaborators or co-design participants and included at all stages, not at the “eleventh hour” before a submission is due. Research is more likely to be relevant, and mobilization is more likely to occur, when it emerges from inclusive and respectful collaboration. This means that stakeholders need to have the ability to guide, shape, and contribute to your grant, research, or paper.
c) **Mobilize knowledge throughout the “life” of your project**. Gone are the days of waiting until a project has finished before sharing findings. There are several actions that should be taken across the life of a project to promote connections, obtain feedback, and generate interactions with your research (19). Examples of this include ongoing conversations and meetings with stakeholders; writing submissions to specific reviews and commissions highlighting your research findings; providing research updates in the form of media releases, media interviews, conference presentations, community presentations, newsletters, and social media posts. For example, according to Altmetric, the capstone “Art dose” paper (3) informing the GAGMH project is in the top 1% of articles by attention internationally and has had 1,116 X (Twitter) posts from 422 X users to an upper bounds of 2,035,515 X followers (25). It is strategic to frame your research in light of current public discourse and provide concise, plain-language summaries of salient research points through the media, social media, blogs, and podcasts. In the last 2 years, GAGMH research has been mobilized via two television interviews (syndicated to five channels nationally), 17 radio interviews (syndicated to 77 stations internationally), and 115 newspaper, magazine, and e-articles.

## Conclusion

While there is no “one-size-fits-all” approach for success in the process of knowledge mobilization, we offer these insights to support the goal of engaging and influencing policy and practice, thus enhancing research impact. Active, meaningful, and respectful conversations, meetings, and collaborations with stakeholders will influence your research framing and process and create the networks and outputs needed to optimize the influence of your research within the complex setting of public health policy and practice.

## Author contributions

CD: Conceptualization, Funding acquisition, Project administration, Supervision, Writing—original draft, Writing—review & editing. MP: Conceptualization, Writing—original draft, Writing—review & editing. SM: Conceptualization, Writing—review & editing. DS: Conceptualization, Writing—review & editing.
